# Post-Stroke Epileptogenesis as a Dynamic Network Process: State-Dependent Biomarkers, Longitudinal Monitoring, and the Concept of an Irritable Brain State

**DOI:** 10.3390/ijms27156822

**Published:** 2026-07-29

**Authors:** Ekaterina Andreevna Narodova

**Affiliations:** Department of Neurology, Prof. V.F. Voyno-Yasenetsky Krasnoyarsk State Medical University, 660022 Krasnoyarsk, Russia; katya_n2001@mail.ru

**Keywords:** post-stroke epilepsy, epileptogenesis, stroke, biomarkers, EEG, neuroinflammation, network reorganization, brain plasticity, longitudinal monitoring, irritable brain state

## Abstract

Post-stroke epilepsy (PSE) is one of the most common long-term neurological complications of stroke and represents a major contributor to disability, reduced quality of life, and increased healthcare burden. Although numerous risk factors and biomarkers have been proposed, many current risk models still emphasize lesion-related predictors and do not fully account for the temporal evolution of post-stroke network excitability. This narrative review aims to examine post-stroke epileptogenesis as a dynamic process of network reconfiguration and to summarize current evidence on biological, neurophysiological, imaging, and clinical biomarkers across different stages following stroke. A structured narrative review of the literature was performed using major biomedical databases. Studies addressing mechanisms of post-stroke epileptogenesis, biomarkers, network dysfunction, neuroinflammation, EEG findings, neuroimaging correlates, and longitudinal monitoring strategies were evaluated and synthesized qualitatively. Evidence suggests that post-stroke epileptogenesis is driven by complex interactions among structural injury, neuroinflammation, blood–brain barrier dysfunction, neurotransmitter imbalance, maladaptive plasticity, and large-scale network reorganization. Importantly, many biomarkers demonstrate substantial temporal variability, and their clinical significance depends on the stage of recovery and the evolving state of brain networks. Emerging data indicate that sleep disturbances, stress, mood disorders, metabolic factors, and other state-dependent influences may further modulate seizure susceptibility after stroke. Post-stroke epileptogenesis may be better conceptualized as a dynamic process of network instability rather than a fixed consequence of focal brain damage. It is proposed that the post-stroke brain can transiently or persistently enter an “irritable brain state”, characterized by increased vulnerability to pathological synchronization under the influence of biological and behavioral modulators. This framework may facilitate the development of longitudinal biomarker strategies and personalized monitoring approaches in future research. However, it should be emphasized that the “irritable brain state” remains a conceptual and hypothesis-generating construct; no validated diagnostic criteria exist, and prospective evidence demonstrating that interventions targeting this state reduce the incidence of post-stroke epilepsy is currently lacking.

## 1. Introduction

Stroke remains one of the leading causes of acquired epilepsy in adults and older individuals worldwide. As advances in acute stroke management continue to improve survival rates, the number of patients living with long-term neurological sequelae has increased substantially. Among these complications, post-stroke epilepsy (PSE) represents one of the most clinically significant outcomes because it is associated with recurrent seizures, impaired functional recovery, reduced quality of life, increased healthcare utilization, and elevated mortality risk [1,2,3,4,5,6,7,8]. The terminology used in this review is consistent with the current ILAE framework for epilepsy definition and classification. For clarity, the following operational endpoints are used throughout this review: acute symptomatic seizures, occurring within 7 days of stroke onset and considered a direct consequence of the acute injury; early seizures, defined here as those occurring within this same ≤7-day window; late seizures, occurring more than 7 days after stroke; and post-stroke epilepsy (PSE), defined as a single late seizure or recurrent unprovoked seizures meeting current ILAE criteria for epilepsy [1,9]. Where the evidence discussed pertains to one of these categories specifically, this is indicated in the corresponding text.

The risk of epilepsy after stroke varies according to stroke subtype, lesion location, lesion volume, age, and the occurrence of acute symptomatic seizures. Previous studies have identified numerous predictors of epileptogenesis, including cortical involvement, hemorrhagic transformation, blood–brain barrier disruption, neuroinflammatory activation, and electroencephalographic abnormalities. Despite considerable progress in identifying these risk factors, the mechanisms linking acute cerebrovascular injury to the later development of epilepsy remain incompletely understood [10,11,12,13,14,15,16,17]. Among ischemic stroke subtypes, cardioembolic and large-artery atherosclerotic strokes are generally associated with greater stroke severity, higher early in-hospital mortality, and less favorable short-term outcomes compared with several other ischemic stroke mechanisms [4,14]. Stroke subtype may also influence the risk of post-stroke epilepsy, although available evidence suggests that this association is mediated largely through factors such as cortical involvement, infarct size, and stroke severity rather than the etiological mechanism alone. Large-artery atherosclerosis is incorporated into the SeLECT score as one of the established predictors of late post-stroke seizures [18].

Traditionally, post-stroke epileptogenesis has often been conceptualized as a relatively direct consequence of structural brain damage. This lesion-centered view, however, leaves a specific knowledge gap unresolved: it does not explain why patients with comparable structural injury frequently follow markedly different clinical trajectories. Addressing this gap requires a conceptual framework capable of capturing processes that unfold after the initial injury rather than being fixed by it. Accumulating evidence increasingly supports such a shift, indicating that the transition from stroke to epilepsy is neither immediate nor uniform. Most patients who experience a stroke never develop epilepsy, while others may develop recurrent seizures months or even years after the initial cerebrovascular event [12,13,16]. This observation indicates that epileptogenesis is likely driven by dynamic biological processes that extend beyond the initial lesion itself.

Experimental and clinical studies increasingly support the view that post-stroke epileptogenesis involves progressive network reorganization occurring over time. Neuroinflammation, alterations in excitatory and inhibitory neurotransmission, blood–brain barrier dysfunction, maladaptive synaptic plasticity, gliosis, and changes in large-scale functional connectivity may interact to influence seizure susceptibility. These mechanisms do not simply co-occur; rather, they are sequentially and reciprocally linked—for example, early neuroinflammation promotes BBB dysfunction, which in turn facilitates excitation–inhibition imbalance and maladaptive plasticity, further amplifying glial dysregulation and network reorganization. This cascade unfolds unevenly across the acute, subacute, and chronic phases following stroke, such that the dominant mechanism, and its clinical significance, may shift over the course of recovery. As a result, the epileptogenic potential of the post-stroke brain may fluctuate over time rather than remain fixed [3,5,14].

This temporal perspective is particularly relevant for biomarker research. Inflammatory mediators, EEG abnormalities, neuroimaging features, and clinical risk scores may have different meanings depending on whether they are assessed during the acute injury phase, the subacute recovery period, or the chronic stage after stroke [18,19,20,21,22]. A marker that reflects tissue damage early after stroke may later indicate persistent network instability or maladaptive remodeling. Therefore, biomarker interpretation should take into account not only the presence of an abnormality but also the stage at which it is detected. However, many studies evaluate such markers at a single time point, despite growing evidence that their predictive value may depend on the evolving biological state of the brain. This single-time-point design is itself a likely source of the inconsistent predictive performance reported across biomarker studies, since a marker’s apparent value may reflect the stage at which it happened to be sampled rather than its intrinsic informativeness. Consequently, static models may not fully capture the dynamic nature of epileptogenesis after stroke, and longitudinal, repeated-measures approaches are needed to determine whether tracking change over time improves prediction beyond single measurements.

Beyond lesion-related mechanisms, several potentially modifiable factors may further influence network excitability during recovery. Sleep disturbances, psychological stress, depression, anxiety, metabolic dysfunction, systemic inflammation, and medication-related effects are common after stroke and may contribute to fluctuations in seizure susceptibility. These observations raise the possibility that post-stroke epileptogenesis reflects not only structural injury but also an evolving interaction between biological, behavioral, and environmental influences.

In recent years, advances in electroencephalography, neuroimaging, digital health technologies, and longitudinal monitoring have provided new opportunities to investigate post-stroke epileptogenesis as a dynamic process rather than a static endpoint. Such approaches may facilitate repeated assessment of network function and improve understanding of why only a subset of stroke survivors ultimately develops epilepsy. This narrative review examines post-stroke epileptogenesis through the perspective of dynamic network reorganization and state-dependent changes in brain excitability. Current evidence regarding biological mechanisms, biomarkers, and longitudinal monitoring approaches is summarized, and temporal fluctuations in network vulnerability that may contribute to epilepsy development after stroke are discussed.

Finally, it is proposed that the post-stroke brain may transiently or persistently enter an “irritable brain state”, characterized by increased susceptibility to pathological synchronization under the influence of evolving biological and behavioral modulators. This framework may provide a useful conceptual perspective for future biomarker development, risk stratification research, and hypothesis generation regarding modifiable mechanisms in post-stroke epilepsy [18,19,20].

This article presents a structured narrative review of current evidence on post-stroke epileptogenesis from the perspective of dynamic network reorganization and state-dependent changes in brain excitability. The review integrates findings from experimental, clinical, neurophysiological, and neuroimaging studies to examine biological mechanisms, biomarkers, and longitudinal monitoring strategies. Given the heterogeneity of the available evidence, the literature was synthesized qualitatively with the aim of developing an integrative conceptual framework rather than performing a systematic review or meta-analysis. The literature search was conducted in PubMed/MEDLINE and Scopus for records published up to June 2026, using combinations of the terms “post-stroke epilepsy,” “post-stroke seizures,” “epileptogenesis,” “biomarkers,” “neuroinflammation,” “blood–brain barrier,” “network reorganization,” and “irritable brain state.” English-language articles were prioritized, with emphasis on studies published within the past 10–15 years alongside foundational earlier work. Given the conceptual and integrative aim of this article, a pre-registered systematic protocol (e.g., PRISMA) was not applied, and eligibility screening was performed by the author based on relevance to the mechanisms and biomarkers under discussion rather than a formal inclusion/exclusion algorithm.

## 2. From Structural Lesion to Dynamic Network Reorganization

The traditional view of post-stroke epilepsy has largely focused on structural brain injury as the principal driver of epileptogenesis. Lesion size, cortical involvement, hemorrhagic transformation, and stroke severity have consistently been associated with an increased risk of subsequent epilepsy. While these factors undoubtedly contribute to epileptogenic processes, they do not fully explain why only a subset of stroke survivors ultimately develops recurrent unprovoked seizures. This discrepancy indicates that lesion anatomy provides only part of the explanation. Two patients with comparable cortical involvement may follow different trajectories: one may recover without seizures, whereas another may develop late unprovoked seizures despite apparent structural stabilization. Such divergence points to post-injury biological and network-level processes that are not captured by lesion size or location alone.

Early post-stroke seizures may also have prognostic significance. Several clinical studies have reported associations between acute symptomatic seizures and worse neurological outcomes, higher in-hospital mortality, and larger infarct burden [23,24]. Because early seizures frequently occur during a period when potentially salvageable peri-infarct tissue remains present, seizure-related increases in metabolic demand may theoretically contribute to secondary neuronal injury and unfavorable clinical outcomes. However, the causal relationship between early seizures and infarct progression remains incompletely established.

Increasing evidence supports the concept that stroke initiates a prolonged process of network reorganization. Rather than representing a static consequence of tissue damage, the post-stroke brain undergoes continuous adaptation involving changes in connectivity, excitability, compensatory plasticity, and large-scale network interactions. These processes begin during the acute phase of injury and may continue for months or even years after the initial cerebrovascular event.

Importantly, network reorganization is not inherently pathological. Many adaptive mechanisms support functional recovery by promoting neuroplasticity, recruitment of alternative pathways, and restoration of disrupted connectivity. However, under certain conditions, the same plastic processes may contribute to maladaptive network remodeling and increased susceptibility to pathological synchronization.

From this perspective, epileptogenesis can be viewed as one possible outcome of post-stroke network evolution rather than as a direct consequence of structural damage. The transition from injury to epilepsy may therefore depend on the balance between stabilizing and destabilizing mechanisms operating within recovering neural networks.

A growing body of evidence suggests that post-stroke networks do not remain in a fixed state. Instead, excitability appears to fluctuate over time under the influence of biological, behavioral, and environmental factors [3,14,25,26]. Neuroinflammatory activity, alterations in excitatory and inhibitory neurotransmission, sleep disturbances, psychological stress, mood disorders, systemic illness, and metabolic changes may all contribute to dynamic shifts in network stability. Consequently, seizure susceptibility may vary considerably throughout the recovery process.

This dynamic perspective provides a potential explanation for several clinical observations. First, epileptic seizures frequently occur months or years after the initial stroke despite apparent stabilization of the structural lesion. Second, many patients with recognized risk factors never develop epilepsy. Third, seizure occurrence may be influenced by factors that are not directly related to lesion characteristics, including sleep deprivation, emotional stress, intercurrent illness, and other state-dependent influences [25,26,27,28,29,30].

Taken together, these observations support a shift from lesion-centered models toward network-centered models of post-stroke epileptogenesis. Within such a framework, stroke may be viewed as the initiating event that triggers a prolonged period of network reconfiguration. During this process, neural systems may fluctuate between relative stability and heightened vulnerability to pathological synchronization.

This evolving vulnerability may represent a clinically relevant intermediate state between structural injury and established epilepsy. Understanding the mechanisms governing transitions between network stability and instability could improve risk stratification, biomarker interpretation, and hypothesis generation regarding future approaches to reducing the long-term burden of post-stroke epilepsy [21,22].

**Figure 1:**
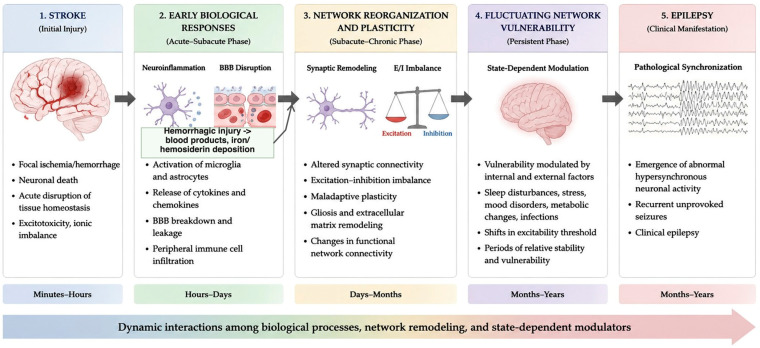
Dynamic evolution of post-stroke epileptogenesis. Stroke initiates a sequence of interacting biological and network-level processes that extend beyond the initial structural injury. Early responses include neuroinflammation and blood–brain barrier (BBB) dysfunction, followed by progressive network reorganization, excitation–inhibition imbalance, and maladaptive plasticity. These processes may result in a state of fluctuating network vulnerability that is further influenced by state-dependent modulators such as sleep disturbances, stress, mood disorders, infections, and metabolic factors. Persistent network instability may ultimately facilitate pathological synchronization and the development of post-stroke epilepsy. The temporal boundaries between stages are approximate and may overlap among individuals. Hemorrhage-specific mechanisms, including blood-product deposition and perihematomal changes, converge on the same early neuroinflammatory stage of this pathway (see Section 3.1).

The proposed sequence of biological and network-level changes contributing to post-stroke epileptogenesis is summarized in Figure 1.

## 3. Biological Drivers of Dynamic Network Vulnerability

Multiple biological processes contribute to the development of post-stroke epilepsy. Traditionally, these mechanisms have often been studied independently, with emphasis placed on specific molecular pathways or structural abnormalities. However, growing evidence suggests that their epileptogenic effects may arise primarily through a common consequence: the progressive alteration of network stability and excitability.

### 3.1. Neuroinflammation and Blood–Brain Barrier Dysfunction

Among the earliest events following stroke is the activation of neuroinflammatory pathways. Both ischemic and hemorrhagic injury trigger a complex inflammatory response involving resident microglia, astrocytes, infiltrating immune cells, and numerous signaling molecules. Although inflammation initially serves protective and reparative functions, persistent or excessive inflammatory activity may contribute to abnormal neuronal excitability and facilitate epileptogenic remodeling [31,32,33,34,35,36].

Hemorrhagic stroke may additionally promote network vulnerability through mechanisms distinct from those of ischemic injury. Extravasated blood products, hemoglobin degradation, and subsequent iron and hemosiderin deposition generate oxidative stress and perilesional tissue injury that can persist long after the acute event, while the evolving perihematomal edema and gliosis further contribute to local network destabilization. These hemorrhage-specific pathways converge on the same downstream processes of neuroinflammation and network reorganization described above, providing a mechanistic rationale for the inclusion of hemorrhagic transformation as a predictor in scores such as CAVE alongside ischemic-stroke-based models such as SeLECT.

Patients with extensive or polyvascular atherosclerotic disease may represent a particularly relevant subgroup in this context. Progression of systemic atherosclerosis has been associated with inflammatory activation and increased risk of recurrent vascular events [37]. Because recurrent cerebrovascular injury itself is a recognized risk factor for post-stroke epilepsy, chronic vascular inflammation may contribute indirectly to persistent network vulnerability and epileptogenic remodeling.

Disruption of the blood–brain barrier (BBB) represents another important consequence of cerebrovascular injury. Experimental and clinical studies indicate that BBB dysfunction may persist beyond the acute phase of stroke and promote abnormal interactions between circulating factors and neural tissue. Such alterations can influence ionic homeostasis, neuroimmune signaling, and synaptic function, thereby creating conditions that favor network instability. In this context, BBB dysfunction is particularly relevant because it provides a mechanistic bridge between vascular injury and neuronal hyperexcitability. Leakage of serum proteins, altered albumin signaling, disturbed potassium buffering, and astrocytic dysfunction may change the extracellular environment in ways that lower the threshold for abnormal synchronization [38,39,40,41,42]. Therefore, BBB disruption should not be interpreted only as a marker of vascular damage but also as a potential driver of persistent network vulnerability [33,34].

### 3.2. Excitation–Inhibition Imbalance

Changes in excitatory and inhibitory neurotransmission also play a central role in post-stroke network dynamics. Acute ischemic injury is associated with excessive glutamatergic activation and excitotoxicity, whereas chronic phases may involve more subtle disturbances in the balance between excitation and inhibition. Importantly, these changes are not necessarily static. The relative contribution of excitatory and inhibitory mechanisms may evolve throughout the recovery process, contributing to temporal fluctuations in seizure susceptibility [3,14,25,26]. Particular attention should be given to inhibitory control mechanisms. Post-injury alterations in GABAergic interneuron function, chloride homeostasis, and glutamate clearance may reduce the capacity of cortical networks to contain excessive excitation. Disturbances involving chloride transporters, potassium-chloride cotransporter 2 (KCC2) and sodium-potassium-chloride cotransporter 1 (NKCC1), may shift GABAergic signaling toward less effective inhibition under certain pathological conditions. Such mechanisms are relevant because even subtle impairment of inhibitory buffering may increase susceptibility to seizure propagation without producing immediate clinical seizures [33,34,35,36,37,38,39,40,41,42]. Beyond the specific mechanisms discussed above, recent conceptual reviews have proposed that alterations in chloride homeostasis should be interpreted within broader frameworks of state-dependent network excitability rather than as isolated molecular abnormalities [43].

### 3.3. Maladaptive Plasticity and Network Connectivity

Stroke additionally induces profound alterations in structural and functional connectivity. Synaptic remodeling, axonal sprouting, dendritic reorganization, and compensatory recruitment of alternative pathways are essential components of recovery. However, these same processes may under certain circumstances facilitate the emergence of hyperexcitable network configurations. Consequently, adaptive and maladaptive plasticity should not be viewed as separate phenomena but rather as different outcomes of the same biological response to injury. A central challenge is that the same plasticity mechanisms supporting post-stroke recovery may, in some patients, promote epileptogenic remodeling. Axonal sprouting, synaptic reweighting, and compensatory recruitment of perilesional or contralesional networks can improve function, but they may also create aberrant excitatory loops or reduce the specificity of network communication [3,44,45]. This dual role of plasticity is particularly important for post-stroke epileptogenesis, where recovery and hyperexcitability may arise from overlapping biological processes.

### 3.4. Glial Contributions to Persistent Network Instability

Recent evidence further highlights the importance of glial cells as active participants in post-stroke epileptogenesis. Beyond their traditional supportive functions, astrocytes and microglia regulate neurotransmitter homeostasis, inflammatory signaling, metabolic support, and synaptic remodeling. Persistent glial activation may therefore contribute to long-term alterations in network behavior even after apparent structural stabilization. Astrocytes are especially relevant in this context because they regulate extracellular potassium, glutamate uptake, water balance, and metabolic support for neurons. After stroke, astrocytic dysfunction may impair these homeostatic functions and thereby promote extracellular conditions favorable to hyperexcitability. Microglia may further shape epileptogenic remodeling through cytokine release, synaptic pruning, and complement-related mechanisms [46,47,48,49,50,51]. Together, astrocytic and microglial responses may help determine whether post-stroke plasticity remains adaptive or evolves toward persistent network instability.

Importantly, these biological mechanisms do not operate independently. Neuroinflammation may exacerbate BBB dysfunction; BBB disruption may influence neurotransmission; altered neurotransmission may affect plasticity; and plastic changes may further modify network organization. The resulting interactions create a dynamic system in which multiple processes converge to influence network excitability over time.

This perspective suggests that no single mechanism is likely to explain post-stroke epileptogenesis in isolation. Rather than acting as independent causal pathways, inflammatory, vascular, neurotransmitter, and plasticity-related processes may collectively shape an evolving state of network vulnerability. The clinical manifestation of this vulnerability depends not only on the presence of individual biological abnormalities but also on their interaction within the broader context of recovering neural systems.

Consequently, post-stroke epileptogenesis may be better understood as an emergent property of dynamic network adaptation. Neuroinflammation, BBB dysfunction, excitation–inhibition imbalance, glial responses, and maladaptive plasticity do not simply add independent risks; they act synergistically, such that their combined effect on network stability exceeds what would be predicted from any single mechanism in isolation. This integrated, mechanism-level picture explains why the post-stroke brain reaches a given level of vulnerability, but it does not yet account for why that vulnerability fluctuates from day to day. The following section addresses this second question by introducing state-dependent modulators that act on top of this biological substrate.

## 4. State-Dependent Modulators of Post-Stroke Network Excitability

Although biological mechanisms such as neuroinflammation, blood–brain barrier dysfunction, and maladaptive plasticity contribute to the development of post-stroke epilepsy, these processes alone may not fully explain the substantial variability observed among patients. Clinical experience demonstrates that seizure susceptibility often fluctuates over time and may be influenced by factors extending beyond the structural lesion and its immediate biological consequences.

This observation suggests that post-stroke network excitability is not solely determined by fixed pathological changes but may also be modulated by dynamic state-dependent influences. Such factors may alter the balance between network stability and instability, thereby affecting the likelihood of seizure generation in vulnerable individuals.

Importantly, these modulators are unlikely to act as primary causes of epileptogenesis. Rather, they may influence an already vulnerable network created by stroke-related injury and remodeling. In this regard, seizure susceptibility may depend not only on the magnitude of structural damage but also on the current physiological state of the recovering brain.

One of the most important modulators of network excitability is sleep. Sleep disturbances are highly prevalent following stroke and include insomnia, sleep fragmentation, excessive daytime sleepiness, circadian rhythm disruption, and sleep-disordered breathing. These abnormalities may influence cortical excitability, synaptic homeostasis, and network synchronization [27,52]. Furthermore, sleep deprivation is a well-established precipitating factor for seizures in various forms of epilepsy and may similarly contribute to seizure susceptibility in post-stroke populations [28]. Sleep-disordered breathing deserves particular attention because obstructive sleep apnea is common after stroke and has been associated with intermittent hypoxia, autonomic dysregulation, systemic inflammation, and alterations in cortical excitability. Although its direct contribution to post-stroke epileptogenesis remains insufficiently studied, sleep apnea may represent a potentially modifiable factor influencing long-term network stability [53].

Psychological stress represents another potentially important determinant of network behavior. Stroke survivors frequently experience chronic stress related to functional impairment, uncertainty regarding recovery, social limitations, and fear of recurrent vascular events. Experimental and clinical studies indicate that stress can influence neuroendocrine signaling, inflammatory pathways, autonomic regulation, and neural network dynamics. While direct evidence linking stress to post-stroke epileptogenesis remains limited, the available data support its potential role as a modulator of network vulnerability [29,30,31,32]. Stress-related effects may be especially relevant during the transition from acute recovery to long-term adaptation. Activation of neuroendocrine pathways, including the hypothalamic–pituitary–adrenal axis, may influence inflammatory responses, synaptic plasticity, and neuronal excitability. Consequently, stress may act not as an isolated trigger but as a factor capable of amplifying pre-existing network vulnerability.

Mood disorders may further contribute to alterations in post-stroke excitability. Depression and anxiety are among the most common neuropsychiatric consequences of stroke and are associated with widespread changes in neurotransmitter systems, sleep architecture, autonomic function, and inflammatory activity [54,55]. Importantly, these factors may interact with existing post-stroke network abnormalities, potentially amplifying susceptibility to pathological synchronization. Notably, depression, anxiety, and sleep disturbances frequently coexist after stroke. Their combined presence may have greater implications for network behavior than any single condition alone. This observation supports the view that seizure susceptibility may emerge from interacting biological and behavioral influences rather than from isolated risk factors.

Systemic physiological conditions may also influence seizure risk after stroke. Intercurrent infections, metabolic disturbances, systemic inflammation, electrolyte abnormalities, and medication-related effects can alter neuronal function and network stability [32,56]. Such factors are frequently encountered during stroke recovery and may contribute to temporal fluctuations in excitability that are not directly reflected by structural imaging findings.

Importantly, these influences rarely occur in isolation. Sleep disruption may increase stress responsiveness; stress may worsen sleep quality; depression may affect both sleep and inflammatory activity; systemic illness may exacerbate fatigue and cognitive dysfunction. The cumulative impact of these interacting factors may substantially exceed the contribution of any single modulator. From a network perspective, these observations suggest that post-stroke epileptogenesis is not exclusively driven by lesion-related pathology. Instead, seizure susceptibility may emerge from the interaction between relatively stable biological alterations (such as persistent BBB dysfunction or established network reorganization) and continuously changing internal influences (sleep quality, mood, and systemic physiological state) and external influences (infection, medication changes, and psychosocial stressors) [27,28,29,30,31,32,52,53,54,55,56,57]. Consequently, the epileptogenic potential of the post-stroke brain may vary across time rather than remain fixed.

This framework may help explain several clinically relevant phenomena, including delayed seizure onset, fluctuating seizure frequency, and the inconsistent predictive performance of many proposed biomarkers. Biomarkers assessed at a single time point may capture only a temporary representation of network status, whereas the underlying vulnerability continues to evolve in response to changing biological and behavioral conditions.

From a conceptual perspective, these observations suggest that post-stroke seizure risk may fluctuate around a moving threshold rather than remain constant throughout recovery. The position of this threshold may be influenced by both relatively stable biological alterations and transient state-dependent modulators. As a result, the same individual may experience periods of relative network stability alternating with periods of increased susceptibility to pathological synchronization.

Taken together, these observations suggest that post-stroke seizure risk should not be interpreted solely through lesion characteristics or isolated molecular pathways. In clinical practice, a vulnerable post-stroke network may be further destabilized by sleep loss, infection, metabolic imbalance, psychological stress, depression, or medication-related factors. Recognizing these modulators may help explain short-term fluctuations in seizure susceptibility and supports the need for repeated, stage-sensitive assessment rather than one-time risk classification.

The principal biological drivers and state-dependent modulators contributing to post-stroke network vulnerability are summarized in Table 1.

Table 1 summarizes the principal biological processes and state-dependent influences discussed in this review. The table illustrates how diverse mechanisms may converge on a common pathway of dynamic network vulnerability, potentially contributing to epileptogenesis after stroke. It should be noted that the strength of supporting evidence varies considerably across these categories: neuroinflammation, BBB dysfunction, and excitation–inhibition imbalance are supported by relatively consistent experimental and clinical data, whereas the contribution of state-dependent modulators such as sleep, stress, and mood disorders is currently supported mainly by indirect or associative evidence. The table should therefore be interpreted as a conceptual summary rather than as an indication of equivalent evidentiary weight across all listed factors. Approximate timelines and, where available, key molecular mediators are provided in the final column to enhance the table’s educational value and to reinforce the manuscript’s central emphasis on the dynamic, time-dependent nature of post-stroke epileptogenesis.

## 5. Biomarkers Across Time: Why Timing Matters

The identification of reliable biomarkers for post-stroke epileptogenesis remains a major challenge in contemporary epileptology. Numerous biological, electrophysiological, imaging, and clinical markers have been proposed to predict seizure risk following stroke. However, despite substantial research efforts, the predictive performance of individual biomarkers has often been inconsistent across studies. Candidate biomarkers of post-stroke epileptogenesis can be broadly categorized into biological, electrophysiological, neuroimaging, and clinical domains. While each category provides valuable information, none appears sufficient when interpreted independently from the temporal stage of recovery. This limitation may partly explain the variability observed across studies investigating predictive markers of post-stroke epilepsy.

One potential explanation for these discrepancies is that most biomarkers are typically assessed as static variables, whereas post-stroke epileptogenesis itself is a dynamic process. Biological mechanisms evolve over time, network organization undergoes continuous adaptation, and seizure susceptibility may fluctuate throughout recovery. Consequently, the significance of a given biomarker may depend not only on its magnitude but also on the temporal context in which it is measured.

This temporal perspective is particularly relevant for inflammatory biomarkers. Abraira et al. demonstrated significant associations between acute blood biomarker profiles—including inflammatory mediators—and subsequent epilepsy development after stroke. However, individual inflammatory markers such as interleukin-1β (IL-1β), interleukin-6 (IL-6), tumor necrosis factor-α (TNF-α), and high-mobility group box 1 protein (HMGB1) have not been shown to have established diagnostic thresholds or consistent predictive accuracy across studies, and no AUC values have been reported for these markers in validated PSE prediction models. The available evidence thus suggests that elevated inflammatory activity is biologically associated with epileptogenic processes, but that the clinical utility of individual inflammatory biomarkers for PSE prediction remains limited by methodological heterogeneity, variability in cohort characteristics, and the absence of standardized sampling protocols [33,35,58,59,60,61]. Importantly, this limitation is consistent with, but does not prove, the hypothesis that the predictive meaning of inflammatory markers may depend on the timing and biological context of assessment. Therefore, longitudinal rather than isolated measurement may ultimately prove more informative, although this assumption requires prospective validation.

The representative biomarker categories, together with the available evidence, temporal characteristics, and major limitations, are summarized in Table 2.

Importantly, the available biomarker literature remains heterogeneous and methodologically limited. Most studies evaluate biomarkers at a single time point rather than examining their longitudinal trajectories throughout the epileptogenic process. In addition, substantial variability exists regarding patient populations, stroke subtypes, timing of sampling, outcome definitions, and duration of follow-up. Inflammatory biomarkers may provide insight into ongoing biological processes associated with epileptogenesis, whereas EEG findings and validated clinical prediction models currently offer more direct clinical risk stratification. However, even established prediction tools such as the SeLECT and CAVE scores are largely based on static assessments and do not explicitly incorporate temporal fluctuations in biological state. Therefore, the present review does not propose that longitudinal biomarkers have already demonstrated superiority over existing clinical prediction models. Rather, it suggests that temporal biomarker dynamics represent a promising but insufficiently studied area that requires prospective validation in dedicated longitudinal studies.

A similar principle may apply to electroencephalographic biomarkers. Early EEG abnormalities often reflect acute cerebral dysfunction and transient network disturbances. In contrast, persistent epileptiform discharges, abnormal rhythmic activity, or prolonged alterations in functional connectivity during later stages may be more closely associated with long-term epileptogenic processes. Consequently, the predictive value of EEG findings may vary according to the timing of assessment. Several electroencephalographic features have been proposed as potential markers of epileptogenic risk, including epileptiform discharges, periodic patterns, focal slowing, abnormal rhythmic activity, and alterations in functional connectivity [62,63,64,65]. Importantly, the prognostic significance of these findings may differ substantially between the acute and chronic phases following stroke.

Neuroimaging biomarkers also demonstrate temporal complexity. Structural imaging performed immediately after stroke primarily characterizes lesion burden and anatomical injury, whereas longitudinal imaging may provide additional information regarding cortical reorganization, network remodeling, secondary degeneration, and compensatory plasticity [41,42,64,65,66,67,68,69]. Such changes may influence epileptogenic risk independently of the original lesion characteristics. Neuroimaging markers may likewise evolve over time. Acute imaging primarily reflects the extent and location of injury, whereas later studies may reveal cortical atrophy, secondary degeneration, network disconnection, altered functional connectivity, and other indicators of long-term remodeling. These longitudinal changes may provide information that is not available from baseline imaging alone.

Importantly, biomarker trajectories may be more informative than isolated measurements. Repeated assessments have the potential to capture evolving biological states and identify individuals whose networks remain unstable despite apparent structural recovery. From this perspective, changes over time may provide clinically relevant information that cannot be obtained from single time-point evaluations. For example, a transient inflammatory response that gradually resolves may indicate successful adaptation and recovery, whereas persistent inflammatory activation, progressive EEG abnormalities, or evidence of ongoing network reorganization may suggest sustained biological vulnerability. Thus, the direction and rate of change may be as informative as the absolute value of a biomarker at any given time. The concept of state-dependent biomarkers offers a useful framework for interpreting these observations. Within this model, biomarkers are not viewed as fixed indicators of future epilepsy but rather as dynamic reflections of ongoing biological and network processes. Their meaning depends on the interaction between the underlying structural and molecular pathology and the timing or physiological context in which the measurement is obtained—that is, the same biomarker value may indicate a different biological reality depending on whether it is assessed during acute injury, ongoing network remodeling, or a period of superimposed state-dependent instability.

This framework may also help explain why some patients with apparently unfavorable biomarker profiles never develop epilepsy, whereas others experience seizures despite relatively modest abnormalities. Biomarkers do not operate independently of the biological context in which they are expressed. Instead, they may represent transient manifestations of evolving network states whose clinical significance changes over time [14,21,22,60,61,62,63,73]. A broader conceptual interpretation of state-dependent biomarker dynamics has recently been proposed in narrative work focusing on neural excitability and epileptogenesis beyond seizure occurrence [72].

Advances in longitudinal monitoring technologies create new opportunities for implementing this perspective in clinical practice. Serial EEG recordings, repeated neuroimaging studies, digital health platforms, wearable devices, and continuous behavioral monitoring may allow clinicians to track biomarker dynamics rather than relying solely on isolated measurements. Such approaches may improve risk stratification and facilitate earlier identification of patients undergoing progressive epileptogenic network reorganization. Viewed collectively, these observations support a transition from biomarker identification toward biomarker dynamics. Rather than asking whether a particular marker predicts epilepsy, future studies may need to determine how biomarker trajectories reflect evolving network states and how these trajectories interact with clinical outcomes. At present, however, evidence demonstrating that longitudinal biomarker assessment improves prediction of post-stroke epilepsy beyond established clinical models remains limited. Taken together, current evidence suggests that the predictive value of biomarkers cannot be fully understood without considering temporal evolution. Post-stroke epileptogenesis unfolds across multiple biological stages, and biomarkers should be interpreted within this dynamic framework. Within this framework, biomarkers may be interpreted as indirect indicators of the current state of network stability rather than as isolated predictors of future disease. This perspective aligns with the concept that epileptogenesis reflects an evolving biological process characterized by changing levels of network vulnerability. Future research may benefit from shifting attention from static predictors toward longitudinal patterns that reflect changing states of network vulnerability and resilience. This perspective naturally supports the use of longitudinal monitoring strategies designed to capture evolving network states over time.

Examples of stage-dependent biomarkers and their potential interpretation across different phases of post-stroke epileptogenesis are summarized in Table 3.

Table 3 illustrates how the biological meaning and clinical relevance of candidate biomarkers may vary across different stages of post-stroke epileptogenesis. The same biomarker may reflect acute injury, ongoing network remodeling, or persistent vulnerability depending on the temporal context in which it is assessed.

It should be emphasized that not all entries in Table 3 represent biomarkers currently suitable for clinical application. Blood–brain barrier markers, glial activation markers, and digital/longitudinal indicators remain largely mechanistic constructs supported by experimental or early translational data, rather than validated, assay-based biomarkers ready for routine clinical use; their inclusion here reflects biological plausibility and research relevance rather than established clinical utility.

## 6. Longitudinal Monitoring and Digital Perspectives

The dynamic nature of post-stroke epileptogenesis has important implications for clinical monitoring. If network vulnerability evolves over time and biomarker significance depends on the stage of recovery, then single-point assessments may provide only limited information regarding future seizure risk. This consideration highlights the potential value of longitudinal monitoring approaches capable of capturing temporal changes in biological and network states.

Current clinical practice often relies on isolated evaluations performed during the acute or early post-stroke period. While these assessments provide important diagnostic and prognostic information, they may not fully reflect the ongoing processes of network reorganization that continue throughout recovery. Consequently, patients with similar findings during the acute phase may subsequently follow markedly different trajectories with respect to epileptogenesis.

Longitudinal electroencephalographic monitoring represents one potential strategy for addressing this limitation. Repeated EEG assessments may provide insight into the evolution of cortical excitability, epileptiform activity, and functional network organization [62,65,74,75,76,77]. Rather than focusing exclusively on the presence or absence of specific abnormalities, serial recordings may help identify persistent or progressive patterns associated with increased epileptogenic risk.

Neuroimaging may offer similar opportunities. Structural and functional imaging performed at multiple time points can capture changes in connectivity, cortical reorganization, and network remodeling that may not be apparent during initial evaluations. Such approaches may improve understanding of how adaptive and maladaptive plasticity contribute to the long-term evolution of seizure susceptibility following stroke.

Beyond conventional clinical tools, digital health technologies have emerged as promising instruments for continuous and patient-centered monitoring [77,78,79,80,81,82]. Mobile applications, wearable devices, remote assessment platforms, and telemedicine systems increasingly enable collection of longitudinal data outside traditional healthcare settings. These technologies may facilitate repeated evaluation of behavioral, physiological, and environmental factors relevant to seizure risk.

Importantly, digital monitoring may be particularly valuable for assessing state-dependent modulators of network excitability. Sleep quality, daily activity patterns, medication adherence, stress levels, mood fluctuations, and other behavioral variables can vary substantially over time and may not be adequately captured during routine clinical visits. Continuous or repeated digital assessment provides an opportunity to evaluate these factors within real-world environments and to examine their relationship with evolving network vulnerability [76,83,84].

The integration of biological, electrophysiological, imaging, and behavioral data may ultimately support a more comprehensive understanding of post-stroke epileptogenesis. Rather than relying on a single biomarker, future monitoring strategies may benefit from combining multiple streams of information to characterize the current state of the recovering brain. Such multidimensional approaches may be particularly useful for identifying patients whose networks remain vulnerable despite apparent clinical stabilization.

Advances in artificial intelligence and machine learning may further enhance these efforts by enabling analysis of complex longitudinal datasets. Although such approaches remain largely investigational in post-stroke epilepsy, they offer the potential to detect patterns that are not readily apparent through conventional clinical assessment. Importantly, future predictive models will likely require validation across diverse populations and clinical settings before widespread implementation.

From a broader perspective, longitudinal monitoring aligns with the concept that epileptogenesis is a process rather than a single event. Repeated assessment may provide greater insight into the evolving balance between recovery, compensation, and network destabilization than isolated measurements obtained at one stage of disease. Such an approach shifts attention from static prediction toward continuous evaluation of changing seizure susceptibility.

Taken together, emerging monitoring technologies support a transition from episodic assessment toward dynamic surveillance of post-stroke network health. By capturing temporal changes in biological, behavioral, and electrophysiological factors, longitudinal monitoring may improve risk stratification, facilitate personalized management, and provide a practical framework for studying the evolving mechanisms of post-stroke epileptogenesis.

## 7. Toward the Concept of an Irritable Brain State After Stroke

The evidence reviewed in the preceding sections suggests that post-stroke epileptogenesis cannot be fully explained by structural injury alone. Although lesion characteristics remain important determinants of seizure risk, they provide only a partial explanation for the substantial heterogeneity observed among stroke survivors. Similarly, individual biological mechanisms, including neuroinflammation, blood–brain barrier dysfunction, neurotransmitter imbalance, and maladaptive plasticity, appear insufficient to account for the dynamic and variable nature of epileptogenesis when considered in isolation.

A common theme emerging from contemporary research is that stroke initiates a prolonged process of network reorganization. During this period, neural systems continuously adapt to injury through mechanisms that may promote either recovery or instability. Importantly, these processes evolve over time and are influenced by numerous biological, behavioral, and environmental factors. As a result, seizure susceptibility appears to fluctuate rather than remain fixed.

This perspective supports a shift from lesion-centered models toward state-oriented interpretations of epileptogenesis. Rather than viewing epilepsy as a direct and inevitable consequence of structural damage, it may be useful to consider the intermediate states through which neural networks pass during recovery. Such states are characterized not only by anatomical abnormalities but also by varying degrees of functional vulnerability to pathological synchronization.

Within this context, the post-stroke brain may transiently or persistently enter a condition of heightened network susceptibility that cannot be fully captured by conventional structural measures alone. For the purposes of this review, this condition may be cautiously conceptualized as an “irritable brain state”, reflecting a reversible or persistent increase in susceptibility to abnormal synchronization under the influence of interacting biological and behavioral modulators. This term is introduced as a heuristic construct intended to facilitate discussion of dynamic epileptogenic processes and should not be interpreted as implying the existence of a distinct pathological state that has been empirically validated.

Importantly, the proposed concept does not imply the existence of a distinct disease entity. The concept of an irritable brain state differs from the traditional notion of a seizure threshold in several respects. Whereas seizure threshold typically refers to a neuronal excitability parameter measured at a specific time point, the irritable brain state represents a dynamic network-level condition shaped by the interaction of biological mechanisms and state-dependent modulators. Unlike fixed thresholds, this state may fluctuate over time and may be at least partially modifiable through interventions targeting sleep, inflammation, or stress. This conceptual distinction aligns with growing evidence that seizure susceptibility in post-stroke populations is not a static biological parameter but an evolving clinical characteristic. Rather, it represents a heuristic framework intended to integrate diverse observations that are often studied separately. Neuroinflammation, disrupted inhibitory control, sleep disturbances, psychological stress, mood disorders, metabolic abnormalities, and evolving network connectivity may all contribute to the emergence and maintenance of this vulnerable state. The relative importance of these factors is likely to vary between individuals and across different stages of recovery.

A key feature of the irritable brain state is its dynamic nature. Network vulnerability may increase or decrease over time depending on the balance between destabilizing and compensatory mechanisms. Consequently, epileptogenesis may be viewed not as a binary transition from stroke to epilepsy but as a continuum characterized by fluctuating levels of seizure susceptibility. Some individuals may progress toward persistent hyperexcitability and epilepsy, whereas others may gradually return to a more stable network configuration. The potential reversibility of the proposed irritable brain state may be conceptually relevant from a clinical perspective. However, direct evidence demonstrating that interventions targeting sleep, inflammation, mood disorders, or other state-dependent factors reduce the incidence of post-stroke epilepsy is currently lacking. Consequently, the present framework should be regarded as a hypothesis-generating model that may help identify future research directions rather than as a validated basis for preventive intervention.

This framework may also provide a useful explanation for several unresolved clinical observations. It may help explain why epilepsy develops only in a subset of patients with apparently similar lesions, why seizures often emerge long after structural stabilization, and why the predictive performance of many biomarkers remains inconsistent across studies. If network vulnerability itself changes over time, then both biomarker expression and clinical risk may vary according to the current biological state of the recovering brain. Importantly, the proposed irritable brain state should not be interpreted as a diagnostic category, clinical syndrome, or established biological entity. At present, no validated biomarkers, objective thresholds, or consensus diagnostic criteria exist that would allow reliable identification of this state in individual patients. The framework is intended primarily as a conceptual model integrating diverse observations from biomarker, neurophysiological, neuroimaging, and clinical studies into a testable hypothesis regarding dynamic network vulnerability after stroke.

Importantly, the concept of an irritable brain state aligns with growing interest in longitudinal monitoring and state-dependent biomarkers. From this perspective, repeated assessment may theoretically provide additional information regarding evolving biological and network states. However, prospective studies comparing longitudinal monitoring with conventional risk assessment approaches are currently limited. Therefore, the potential clinical value of repeated assessment remains to be established.

Although the present framework remains conceptual and requires further empirical validation, it offers a potential bridge between molecular mechanisms, network neuroscience, biomarker research, and clinical observation. By emphasizing dynamic vulnerability rather than static pathology, it may provide a useful perspective for future studies investigating the mechanisms, prediction, and prevention of post-stroke epilepsy.

**Figure 2:**
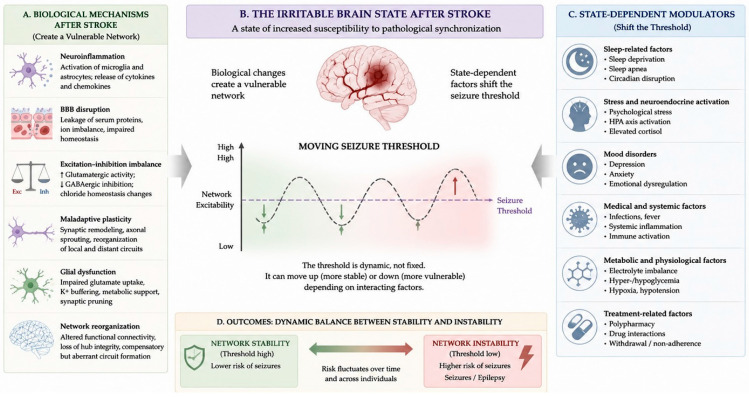
The irritable brain state after stroke. Post-stroke epileptogenesis may be viewed as a dynamic interaction between biological mechanisms that create a vulnerable network and state-dependent modulators that influence seizure susceptibility over time. Neuroinflammation, blood–brain barrier dysfunction, excitation–inhibition imbalance, maladaptive plasticity, glial activation, and network reorganization contribute to the formation of a vulnerable neural state. Sleep disturbances, stress, mood disorders, systemic illness, metabolic abnormalities, and treatment-related factors may subsequently shift the seizure threshold, resulting in fluctuations between relative network stability and increased vulnerability to pathological synchronization. The model illustrates post-stroke epileptogenesis as a dynamic process rather than a fixed consequence of structural brain injury. Arrows indicate the proposed direction of interactions and dynamic relationships between biological mechanisms, state-dependent modulators, and the evolving irritable brain state.

The conceptual framework of the proposed irritable brain state after stroke is illustrated in Figure 2.

The irritable brain state, as proposed here, should be distinguished from several related but conceptually distinct constructs. The epileptogenic network concept and staged models of latent epileptogenesis [3] describe the structural and molecular evolution of tissue toward seizure capability, typically over a single, unidirectional trajectory; the irritable brain state instead emphasizes bidirectional, reversible fluctuations in seizure threshold superimposed on this trajectory. Seizure threshold variability is a well-established electrophysiological phenomenon, but is not itself tied to a specific post-injury mechanism or population. Cyclical and proictal-state models of seizure risk, developed primarily in chronic drug-resistant epilepsy through long-term intracranial or sub-scalp EEG monitoring, describe recurring, often circadian or multi-day periodicity in seizure probability [76,83,84]. The irritable brain state framework proposed here is intended to be complementary to, rather than a replacement for, these models: it specifically addresses the post-stroke recovery period, in which network vulnerability is shaped by the interaction of an evolving structural/molecular substrate with superimposed, often non-periodic behavioral and systemic modulators.

## 8. Clinical and Translational Implications

The dynamic framework proposed in this review has several potential implications for clinical practice and future research. Traditionally, risk assessment in post-stroke epilepsy has focused primarily on relatively stable factors such as lesion characteristics, stroke subtype, and the presence of early seizures. While these variables remain important, the evidence reviewed here suggests that epileptogenic risk may continue to evolve long after the initial cerebrovascular event.

One practical consequence of this perspective is the recognition that seizure susceptibility may not be adequately represented by a single assessment performed during the acute phase of stroke. Patients with apparently similar clinical profiles may follow substantially different trajectories depending on the subsequent evolution of biological and network-level processes. Consequently, longitudinal evaluation may represent a promising approach for future investigation. However, current evidence remains insufficient to conclude that repeated assessment provides superior risk prediction compared with established clinical models.

The proposed framework also highlights the importance of potentially modifiable factors that influence network excitability during recovery. Sleep disturbances, mood disorders, psychological stress, systemic inflammation, metabolic abnormalities, and medication-related effects are common after stroke and may contribute to fluctuations in network stability. Although direct evidence for preventive interventions remains limited, recognition and management of such factors may represent an additional component of comprehensive post-stroke care [25,26,27,28,29,30,49,50,51,52,53].

From a biomarker perspective, the concept of dynamic network vulnerability suggests that future predictive strategies should extend beyond static risk models. Repeated assessment of electrophysiological, imaging, biological, and behavioral markers may improve identification of individuals undergoing progressive epileptogenic reorganization. Such approaches may be particularly relevant for patients with persistent neurological symptoms, recurrent transient events, unexplained cognitive fluctuations, or evolving electroencephalographic abnormalities.

The framework presented here may also facilitate integration between traditionally separate research domains. Molecular studies investigating neuroinflammation, neuroimaging research exploring network reorganization, electrophysiological investigations of cortical excitability, and digital health approaches to longitudinal monitoring all address different aspects of the same evolving process. A state-oriented perspective may therefore provide a common conceptual language linking these fields.

Emerging digital technologies offer additional opportunities for translational application. Mobile health platforms, wearable sensors, telemedicine systems, and remote monitoring tools may enable repeated assessment of behavioral and physiological variables relevant to network stability. Such technologies could ultimately support individualized surveillance strategies and facilitate earlier recognition of patients at increased risk of developing epilepsy following stroke.

Importantly, the proposed framework should not be interpreted as a replacement for established clinical risk factors or existing diagnostic approaches. When post-stroke epilepsy becomes established, standard epilepsy terminology, including the definition of drug-resistant epilepsy, remains relevant for treatment escalation and long-term management [85]. Rather, it complements current models by emphasizing that epileptogenic risk may be influenced by ongoing interactions among structural injury, biological mechanisms, and state-dependent modulators. Incorporating these dynamic processes into future predictive models may improve both mechanistic understanding and clinical decision-making.

Ultimately, viewing post-stroke epileptogenesis as a process of evolving network vulnerability may encourage a transition from static prediction toward continuous risk assessment. Such an approach has the potential to support more personalized monitoring strategies and may help identify future opportunities for epilepsy prevention following cerebrovascular injury.

## 9. Limitations and Future Directions

Several limitations should be considered when interpreting the framework proposed in this review.

First, much of the evidence discussed originates from heterogeneous sources, including clinical studies, experimental investigations, neuroimaging research, electrophysiological analyses, and translational models. Although these data collectively support the concept of dynamic network vulnerability following stroke, direct evidence specifically linking all proposed mechanisms within a single longitudinal framework remains limited. It should also be acknowledged that, because this is a narrative rather than a systematic review, source selection and synthesis were not governed by a pre-registered protocol such as PRISMA, and therefore carry an inherent risk of subjective emphasis in the choice and interpretation of the literature discussed. In addition, the evidence linking specific state-dependent modulators (sleep disturbances, psychological stress, and mood disorders) to post-stroke epileptogenesis is, at present, largely associative rather than causal; most supporting studies are small, methodologically heterogeneous, and were not designed to test causal pathways, and aggregate quantitative estimates of predictive performance (e.g., pooled odds ratios or AUC values) across this body of evidence are not currently available. These considerations should be taken into account when interpreting the strength of the conclusions drawn in this review. Second, the concept of an irritable brain state should be regarded as a heuristic and integrative model rather than a formally validated biological construct. At present, no universally accepted diagnostic criteria or objective thresholds exist for defining such a state. The framework is intended primarily to facilitate interpretation of diverse findings and to generate testable hypotheses for future research. To avoid becoming a purely explanatory construct, the irritable brain state framework should generate specific, falsifiable predictions—for example, that repeated biomarker measurements obtained during a defined high-risk window (e.g., following an intercurrent infection or a period of severe sleep disruption) should show a measurable, transient shift toward a pro-epileptogenic profile compared with measurements obtained during a stable interval in the same patient; failure to detect such within-subject state-dependent shifts would argue against the construct’s validity. Third, post-stroke epilepsy is itself a heterogeneous condition. Differences in stroke subtype, lesion location, lesion volume, age, comorbidities, genetic background, and treatment strategies may substantially influence epileptogenic trajectories. Consequently, the relative contribution of biological and state-dependent factors is likely to vary across patient populations.

Sex-related differences were not a primary focus of the present review. However, biological sex may influence stroke characteristics, vascular risk factor profiles, clinical outcomes, and potentially post-stroke epileptogenesis. This area remains insufficiently studied and warrants further investigation in future longitudinal studies. Another important limitation concerns biomarkers. Although numerous candidate biomarkers have been proposed, their predictive value remains inconsistent across studies. The present review argues that temporal variability may contribute to these inconsistencies; however, this hypothesis requires prospective validation using standardized longitudinal protocols.

Future research should focus on characterizing the temporal evolution of post-stroke network vulnerability through repeated multimodal assessments. Longitudinal studies integrating electroencephalography, neuroimaging, biological markers, behavioral measures, and digital monitoring technologies may provide a more comprehensive understanding of epileptogenic processes than currently available approaches. Finally, this review has focused primarily on mechanisms and biomarkers rather than on interventions; existing trials targeting potentially modifiable factors such as sleep-disordered breathing, post-stroke depression, or systemic inflammation were not systematically reviewed here, and their efficacy in reducing post-stroke epilepsy risk specifically has not been established. Prospective interventional studies designed to test whether correcting these modifiable state-dependent factors reduces epileptogenic risk represent an important direction for future research. Future studies should also investigate post-stroke epileptogenesis in very old patients, a rapidly growing population with distinct vascular risk profiles, comorbidity burden, functional trajectories, and competing mortality risks [86]. Whether biomarker dynamics, biomarker interpretation, and network vulnerability evolve differently in this age group compared with younger stroke survivors remains largely unknown and represents an important direction for future research.

Particular attention should be directed toward identifying biomarkers capable of tracking changes in network stability over time rather than merely predicting outcomes from single measurements. Such markers may improve risk stratification and facilitate earlier recognition of individuals progressing toward epilepsy.

Finally, the concept of an irritable brain state may offer a useful framework for future investigation, but its clinical value remains to be established. Prospective studies are required to determine whether dynamic assessment of network vulnerability can improve prediction, prevention, or management of post-stroke epilepsy. Until such evidence becomes available, the proposed model should be considered hypothesis-generating rather than definitive.

### Knowledge Gaps

Despite growing interest in post-stroke epileptogenesis as a dynamic network process, several critical knowledge gaps remain unaddressed in the current literature. First, no validated longitudinal biomarker protocol exists for systematic tracking of network vulnerability across the full recovery trajectory after stroke. Most available studies evaluate candidate biomarkers at a single time point, leaving the temporal dynamics of epileptogenic processes poorly characterized. Second, the relative contribution of state-dependent modulators—including sleep disturbances, psychological stress, mood disorders, and systemic inflammation—to individual epileptogenic trajectories has not been prospectively quantified. These factors are commonly encountered in stroke survivors but are rarely incorporated into biomarker studies or risk prediction models. Third, the proposed concept of an irritable brain state currently lacks operational diagnostic criteria and objective biomarker thresholds, limiting its direct translational applicability. Prospective studies are required to determine whether this framework can be operationalized into clinically useful monitoring or intervention strategies. Fourth, sex-related differences in post-stroke epileptogenesis remain insufficiently characterized. Biological sex may influence vascular risk profiles, inflammatory responses, and network plasticity after stroke, yet existing cohort studies rarely report sex-stratified analyses. Fifth, very old patients aged 85 years and older are systematically underrepresented in existing research, despite representing a rapidly growing population with distinct comorbidity burden and functional trajectories. Whether biomarker dynamics and network vulnerability evolve differently in this age group compared with younger stroke survivors remains largely unknown. Addressing these gaps through dedicated longitudinal studies with standardized multimodal assessment protocols represents a priority for future research in post-stroke epilepsy.

## 10. Conclusions

Post-stroke epilepsy represents one of the most clinically significant long-term complications of cerebrovascular disease, associated with recurrent seizures, impaired functional recovery, reduced quality of life, and increased healthcare burden. The present narrative review examined post-stroke epileptogenesis from the perspective of dynamic network reorganization and state-dependent modulation of brain excitability.

The evidence reviewed across Section 3 and Section 4 consistently supports the view that post-stroke epileptogenesis is driven by complex interactions among structural injury, neuroinflammation, blood–brain barrier dysfunction, excitation–inhibition imbalance, maladaptive plasticity, and glial dysregulation [3,4,5,6,7,14,15,25,26]. These mechanisms do not act as independent causal pathways but converge over time to shape an evolving state of network vulnerability that extends well beyond the initial cerebrovascular event.

Section 5 identified sleep disturbances, psychological stress, mood disorders, systemic inflammation, metabolic abnormalities, and medication-related factors as state-dependent modulators capable of further influencing network excitability during recovery. These factors are common after stroke, interact with one another, and may contribute to temporal fluctuations in seizure susceptibility that are not captured by lesion-based risk models alone.

The biomarker evidence reviewed in Section 6 demonstrated that the predictive value of inflammatory markers, EEG findings, neuroimaging features, and clinical prediction scores depends substantially on the temporal context of assessment. Established models such as the SeLECT and CAVE scores provide useful risk stratification but are based on static parameters. Longitudinal biomarker assessment may offer additional insight into evolving network states, although prospective evidence demonstrating superiority over existing clinical models is currently lacking.

Section 7 outlined emerging opportunities for longitudinal monitoring through serial EEG recordings, repeated neuroimaging, digital health platforms, and wearable technologies. These approaches may facilitate repeated evaluation of biological and behavioral variables relevant to seizure risk beyond the acute phase of stroke.

Based on the integrated evidence reviewed, Section 8 proposed that the post-stroke brain may transiently or persistently enter an irritable brain state—a condition of heightened susceptibility to pathological synchronization arising from the interaction of structural injury, biological mechanisms, and state-dependent modulators. This framework is offered as a hypothesis-generating conceptual model linking molecular, network, and clinical observations. It should not be interpreted as a validated diagnostic entity, and no evidence-based clinical recommendations can currently be derived from it.

Taken together, the findings of this review support a transition from lesion-centered toward network-oriented and state-sensitive models of post-stroke epileptogenesis [3,4,5,6,7,14,15,25,26]. Future prospective studies with standardized longitudinal protocols, multimodal biomarker assessment, and diverse patient populations are required to determine whether dynamic evaluation of network vulnerability can improve prediction, monitoring, and ultimately prevention of post-stroke epilepsy.

## Figures and Tables

**Table 1:** Biological drivers and state-dependent modulators of post-stroke network vulnerability, summarized from the literature discussed in Section 3, Section 4 and Section 5.

Domain	Mechanism/Factor	Potential Contribution to Network Vulnerability	Key Molecules/Pathways and Approximate Timeline
**Biological Drivers**	Central/local neuroinflammation (within injured brain tissue)	Promotes neuronal hyperexcitability, cytokine-mediated network destabilization, and epileptogenic remodeling	IL-1β, IL-6, TNF-α, HMGB1; microglial/astrocytic activation and cytokine release; acute–subacute phase (hours to weeks post-stroke)
	Blood–brain barrier dysfunction	Alters ionic homeostasis, neuroimmune signaling, and extracellular environment	Albumin extravasation, disrupted potassium buffering, astrocytic end-foot dysfunction; acute phase with potential persistence into the subacute period (hours to weeks)
	Excitation–inhibition imbalance	Reduces inhibitory buffering and facilitates abnormal synchronization	Glutamatergic excitotoxicity (acute phase); GABAergic interneuron dysfunction and altered chloride homeostasis via KCC2/NKCC1 cotransporters (subacute–chronic phase); days to months
	Maladaptive plasticity	Supports formation of hyperexcitable circuits and aberrant connectivity	Axonal sprouting, synaptic reweighting, aberrant excitatory circuit formation; subacute–chronic phase (weeks to months)
	Glial dysfunction	Impairs glutamate clearance, potassium buffering, metabolic support, and synaptic regulation	Astrocytic dysregulation of extracellular potassium, glutamate uptake, and water balance; microglial cytokine release, synaptic pruning, and complement-related signaling; subacute–chronic phase (weeks to months, may persist into chronic phase)
	Network reorganization	Alters functional connectivity and may facilitate pathological synchronization	Altered functional/structural connectivity, changes in large-scale network topology; subacute to chronic phase (months, may extend to years)
**State-Dependent Modulators**	Sleep disturbances	Influence cortical excitability, synchronization, and seizure threshold	Variable; may emerge acutely and persist into the chronic phase
	Sleep-disordered breathing	Contributes to intermittent hypoxia, autonomic dysregulation, and inflammation	Often chronic; may emerge subacutely and persist long-term
	Psychological stress	Modulates neuroendocrine pathways, inflammatory activity, and excitability	Subacute to chronic phase; often related to functional impairment during recovery
	Depression and anxiety	Affect neurotransmission, sleep architecture, and network regulation	Subacute to chronic phase; commonly emerge weeks to months after stroke
	Systemic inflammation and infection (peripheral trigger, distinct from central injury response)	Increase biological stress and may transiently lower seizure threshold	Episodic; may occur at any stage of recovery
	Metabolic disturbances	Alter neuronal function through electrolyte or metabolic imbalance	Episodic; may occur at any stage of recovery
	Medication-related factors	Influence excitability through interactions, withdrawal, or non-adherence	Variable; dependent on treatment timing and adherence
**Clinical Consequence**	Dynamic network vulnerability	Fluctuating susceptibility to pathological synchronization and seizure generation	Reflects the cumulative timeline of the preceding biological drivers and state-dependent modulators; typically becomes clinically relevant from the subacute phase onward (weeks to years)
**Potential Outcome**	Persistent instability	Development of post-stroke epilepsy	Chronic phase (months to years); represents unresolved network vulnerability culminating in post-stroke epilepsy
	Network stabilization	Functional recovery without epilepsy	Variable; may occur at any stage of recovery if adaptive mechanisms predominate over destabilizing processes

**Table 2:** Representative biomarker categories in post-stroke epileptogenesis: evidence, timing, and limitations.

Biomarker Category	Representative Studies	Timing After Stroke	Representative Sample(s)	Main Findings	Predictive Performance (If Reported)	Major Limitations
Inflammatory biomarkers (IL-1β, IL-6, TNF-α)	Abraira et al. [60,61] (PSE)	Acute/subacute phase (hours to days after stroke onset)	Final analyzed cohort: *n* = 895 [60]; *n* = 974 [61]	Elevated acute-phase inflammatory markers associated with increased risk of post-stroke epilepsy; Abraira et al. reported significant associations between blood biomarker profiles and epilepsy development, although individual marker predictive accuracy was not systematically quantified	No AUC or diagnostic threshold values established; associations reported without standardized predictive performance metrics	Heterogeneous sampling timepoints, absence of standardized concentration thresholds, single time-point assessment, and lack of validated diagnostic cut-off values for individual inflammatory markers.
HMGB1	Experimental and clinical studies [58,59] (mechanistic association; no single defined clinical endpoint)	Acute/subacute phase (hours to several days after stroke onset)	Not applicable (narrative reviews summarizing experimental and small clinical studies; no single patient cohort).	Increased levels associated with neuroinflammation and epileptogenic processes	Not established	Limited clinical validation and absence of standardized cut-off values
EEG abnormalities (epileptiform discharges, periodic patterns)	Schubert et al.; Bentes et al.; Abe et al.; Fukuma et al. [62,63,64,65] (PSE/late seizures)	Early post-stroke period (within 24 h to 2 weeks after stroke onset)	Schubert et al.: *n* = 1189 with EEG within 7 days after stroke, including derivation cohort *n* = 980 and external validation cohorts *n* = 125 and *n* = 84 [63]; Bentes et al.: *n* = 151 [62]; Abe et al.: NCVC cohort *n* = 187 and validation cohort *n* = 187 [64]; Fukuma et al.: *n* = 211 [65].	Presence of epileptiform discharges and periodic patterns associated with increased risk of post-stroke epilepsy; the updated SeLECT-EEG model demonstrated improved predictive accuracy over the original clinical model	SeLECT-EEG: C-statistic 0.75 (95% CI 0.71–0.80) vs. 0.71 (95% CI 0.65–0.76) for SeLECT 2.0 in stroke survivors without acute symptomatic seizures [63].	Inter-rater variability in EEG interpretation, heterogeneity in timing and duration of EEG recording, limited standardization of EEG protocols across centres
Neuroimaging markers	Boot et al. [44]; Wu et al. [45]; Mishra et al.; Reddy et al.; van Vliet et al. [66,67,68,69,70,71,72] (PSE)	Acute phase (within 24–72 h) and chronic phase (months to years after stroke)	Boot et al.: *n* = 51, including 23 participants with post-stroke epilepsy and 28 without post-stroke epilepsy [44]; remaining evidence derives from narrative reviews, experimental studies, and heterogeneous observational cohorts without a single pooled patient population.	Cortical involvement and large lesion volume consistently associated with increased PSE risk; lesion-to-cortex ratio and perilesional network disruption identified as additional structural predictors; altered functional connectivity on advanced imaging associated with maladaptive network remodeling	Cortical involvement: OR approximately 2.0–4.5 across cohort studies; no unified AUC established for imaging biomarker combinations; predictive performance varies substantially by imaging modality, timing, and outcome definition	Methodological heterogeneity across studies, lack of standardized imaging protocols, limited longitudinal validation, and absence of prospective multimodal imaging studies specifically designed for PSE prediction
SeLECT score	Galovic et al. [18] (late seizures)	Early post-stroke phase (assessment within the first days to weeks after stroke onset)	*n* = 1395 (derivation and validation cohorts combined)	Clinical prediction model integrating stroke severity, large-artery atherosclerosis, early seizures, cortical involvement, and MCA territory infarction	Good predictive performance (AUC approximately 0.77 in derivation and validation cohorts)	Static model primarily based on baseline clinical characteristics
CAVE score	Haapaniemi et al. [19] (late seizures, post-ICH)	Early post-hemorrhagic phase (assessment within the first days after intracerebral hemorrhage onset)	*n* = 357	Clinical risk stratification following intracerebral hemorrhage	Moderate predictive performance (AUC approximately 0.73–0.76 in original cohorts)	Applicable primarily to intracerebral hemorrhage populations

**Table 3:** Stage-specific biomarkers across acute, subacute, and chronic phases of post-stroke epileptogenesis, summarized from the literature discussed in Section 6.

Biomarker Domain	Acute Phase (Hours–Days)	Subacute Phase (Days–Weeks)	Chronic Phase (Months–Years)	Potential Relevance to Epileptogenesis
Inflammatory biomarkers	IL-1β, IL-6, TNF-α, HMGB1 elevation reflecting tissue injury	Persistent inflammatory activation	Chronic low-grade neuroinflammation	Sustained inflammatory signaling may promote network instability
Blood–brain barrier markers	Acute BBB disruption, albumin extravasation	Ongoing barrier dysfunction	Persistent BBB permeability in selected patients	Facilitates abnormal neuroimmune interactions and hyperexcitability
EEG abnormalities	Focal slowing, diffuse dysfunction, acute epileptiform patterns	Persistent epileptiform discharges, abnormal rhythmic activity	Stable epileptiform activity, altered network synchronization	Reflect evolving cortical excitability and seizure susceptibility
Structural neuroimaging	Lesion volume, cortical involvement, hemorrhagic transformation	Early reorganization and peri-lesional changes	Cortical atrophy, secondary degeneration	May influence long-term epileptogenic risk
Functional neuroimaging	Acute network disruption	Connectivity reorganization	Persistent network disconnection or maladaptive connectivity	Reflects network-level remodeling
Glial activation markers	Microglial and astrocytic activation	Ongoing neuroimmune signaling	Chronic glial dysregulation	Contributes to persistent hyperexcitability
Sleep-related markers	Acute sleep disruption during hospitalization	Persistent insomnia or sleep fragmentation	Chronic sleep disorders, sleep apnea	May lower seizure threshold and modulate network vulnerability
Behavioral and psychological factors	Acute stress response	Depression, anxiety, stress-related adaptation	Chronic psychological burden	May influence state-dependent seizure susceptibility
Digital and longitudinal indicators	Baseline physiological and behavioral assessment	Tracking recovery trajectories	Long-term monitoring of network vulnerability	May identify evolving risk patterns over time

## Data Availability

No new data were created or analyzed in this study. Data sharing is not applicable to this article.
